# The Development of a Phytopathogenic Fungi Control Trial: *Aspergillus flavus* and *Aspergillus niger* Infection in Jojoba Tissue Culture as a Model

**DOI:** 10.1155/2021/6639850

**Published:** 2021-01-17

**Authors:** Nawal Abd El-Baky, Raoufa Ahmed Abdel Rahman, Mona Mohammed Sharaf, Amro Abd Al Fattah Amara

**Affiliations:** ^1^Protein Research Department, Genetic Engineering and Biotechnology Research Institute, City of Scientific Research and Technological Applications, Alexandria, Egypt; ^2^Pharmaceutical Bio-Products Research Department, Genetic Engineering and Biotechnology Research Institute, City of Scientific Research and Technological Applications, Alexandria, Egypt

## Abstract

After introducing the idea of using concentrations equal to or less than the minimum inhibition concentration (MIC) of some active chemical compounds for evacuating microbial cells, different types of microbes were evacuated. The original protocol was given the name sponge-like protocol and then was reduced and modified from a microorganism to another to prepare microbial ghosts for various applications such as immunological applications, drug delivery, and isolation of DNA and protein. Fungal pathogens that infect plants critically affect cost effectiveness, quality, and quantity of their production. They kill plant cells and/or cause plant stress. Plant fungal infections can originate from many sources such as infected soil, seeds, or crop debris causing diseases and quality losses around the world with billions of US dollars annually as costs of the associated productivity loss. This study focused on the application of the sponge-like protocol in protecting *in vitro* tissue cultures of plants against fungal pathogens. This can be useful for research purposes or may be developed to be introduced in field applications. *Aspergillus flavus* and *Aspergillus niger* infection in tissue culture of jojoba (*Simmondsia chinensis* (Link) Schn.) was used as a model to establish the employment of this protocol to control plant fungal diseases. The best conditions for *A. flavus* and *A. niger* ghosts production previously mapped by randomization experimental design (reduced Plackett–Burman experimental design) were used to prepare fungal ghosts. SDS, NaOH, NaHCO_3_, and H_2_O_2_ were used in their MIC (+1 level) or minimum growth concentration (MGC, −1 level) according to the determined optimal experimental design. The release of both of DNA and protein from the fungal cells was evaluated spectrophotometrically at 260_nm_ and 280_nm_, respectively, as an indicator for cell loss of their cytoplasm. Fungal ghost cells were also examined by transmission electron microscopy. After confirming the preparation of high-quality fungal ghost cells, the same conditions were mimicked to control plant fungal infection. Jojoba grown in tissue culture was sprayed with fungal cells (about 10^3^ CFU) as a control experiment or fungal cells followed by treatment with solution (a) represents the fungal ghost cells formation calculated critical concentration (FGCCC) of SDS, NaOH, and NaHCO_3_ and then treatment with solution (b) represents H_2_O_2_ FGCCC. The plant was examined on day 0 (plant grown before any infection or infection followed by treatment), day 5 (plant at day 5 after infection or infection followed by treatment), and day 10 (plant at day 10 after infection or infection followed by treatment). We observed fungal growth in case of control experiments at days 5 and 10 on the tissue culture medium, as well as plant, and the absence of any fungal growth in case of plant treated with FGCCC even after day 10. We recommend using this FGCCC in the form of chemical spraying formulation to treat the plants aiming to control different plant fungal infections in *in vitro* tissue culture systems or applied in field.

## 1. Introduction

Phytopathogenic fungi cause most of the diseases arising in agricultural and horticultural setups [1]. Generally, phytopathogens developed infection strategies to attack any plant [2], searching for entry and sourcing nutrients forcefully to achieve microbial growth and development [3]. These pathogens can overcome plant immune defences [4, 5]. This negatively affects the health, homeostasis, and physiology of plants and may result in systemic damage [6]. Plant fungal pathogens can be responsible for a complexity of problems for farmers in plant production as listed by Dean et al. [7] who reviewed the top ten plant fungal pathogens. To overcome the problem of persistent attack of phytopathogenic fungi on agricultural products, numerous agrochemicals were used, some of which are toxic to humans and a withdrawal period is essential between the last dosage and crops harvesting. Besides, they may also have negative environmental effects on soil organisms, insects, and plant pollinators [8].

*Aspergillus niger* (black mold) is one of the most common fungal species of the genus *Aspergillus*. It is abundant in soil and common in indoor environments [9]. It shows phytopathogenic effects [10] causing black mold disease on certain fruits and vegetables and is a common contaminant of food. *Aspergillus niger* is responsible for sooty mold of onions and ornamental plants. *Aspergillus flavus* (yellow mold) exists worldwide as a soil saprobe and infects various important agriculture crops. Common hosts of the pathogen are legumes, cereal grains, and tree nuts [11]. This pathogen can produce the polyketide-derived carcinogenic and mutagenic secondary metabolite, which is called aflatoxin [12, 13]. Aflatoxin causes aflatoxicosis due to inhaling or ingesting food and feed contaminated with high levels of aflatoxin.

Amara et al. [14] were the first to introduce and develop the MIC-MGC chemical technique to gently induce pores in the microbial cells. They named the new technique as sponge-like protocol for preparing bacterial ghosts. The protocol is based on using MIC-MGC of a combination of some cheap, safe, and active chemical compounds (SDS, NaOH, NaHCO_3_, and H_2_O_2_) for evacuating microbial cells. The Plackett–Burman optimization and randomization experimental design (full and reduced) was applied to map the best chemical and physical conditions to produce bacterial ghosts [14–16]. The protocol enables evacuation of microbial cells while keeping their 3D structure. This turns live cells to dead ghost cells with retained immunogenicity that could be used in immunization [17, 18]. Microbial ghosts prepared by this protocol can also be applied as a drug delivery system [19]. Even the cytoplasm itself released from the evacuated ghost cells contains many important biomacromolecules and biological structures other than DNA and protein. The sponge-like chemical protocol was evolved to produce microbial ghosts from Gram-negative [14–17, 20–22] and Gram-positive [23, 24] bacteria, *Saccharomyces cerevisiae*, and *Candida*, filamentous fungi [25, 26].

In this study, as an approach to develop the benefits of the sponge-like chemical protocol, it was applied to protect *in vitro* tissue cultures of plants against fungal pathogens. As a model, tissue culture of jojoba *(Simmondsia chinensis* (Link) Schn.) was infected with *A. flavus* and *A. niger* representing the phytopathogenic fungi. The infected plant tissue culture was sprayed with FGCCC for each fungus. The fungal growth was observed after 5 and 10 days of infection followed by treatment. The success of the protocol to prevent plant fungal infection can be useful for research purposes or may be developed to be introduced in field applications.

## 2. Materials and Methods

### 2.1. Fungal Strains

*A. flavus* and *A. niger* strains used in this study were kindly identified and provided by the Al-Azhar University Mycology Center (Cairo, Egypt).

### 2.2. Cultivation Conditions

*A. flavus* and *A. niger* strains were cultivated in one-litre shake flasks containing 500 ml Sabouraud's dextrose broth at 28°C for 7 days at 150 rpm.

### 2.3. FGCCC

The MIC and MGC values for NaOH, SDS, NaHCO_3_, and H_2_O_2_ against *A. flavus* and *A. niger* were determined as previously reported [25, 26]. The best conditions for *A. flavus* and *A. niger* ghosts production were formerly mapped by randomization experimental design (reduced Plackett–Burman experimental design) [25, 26] and then used to prepare fungal ghosts. The different variables were four chemical compounds: NaOH, SDS, NaHCO_3_, and H_2_O_2_. The four variables were adjusted according to the optimal experimental design for each fungus as described in Table 1.

Each variable of the four used chemical compounds was represented at two levels (high and low), which are donated by +1 (MIC) and −1 (MGC), as in Table 1 (except that SDS was used in only the +1 level and NaOH was used in only the −1 level).

The fungal biomass of the cultivated fungi at 25°C for 7 days was collected, washed gently by 0.5% saline, and then, recentrifuged at 6000 rpm for 10 min. The supernatant was discarded afterwards. 5X stock solution for each of NaOH, SDS, and NaHCO_3_ and 2X stock solution for H_2_O_2_ were prepared from both +1 (MIC) and −1 (MGC) levels according to Table 1 (except SDS and NaOH were used in only the +1 and −1 level, respectively). All stock solutions were filter sterilized by 0.22 *μ*m TPP syringe filter (St. Louis, Mo., USA).

Fungal ghost cells preparation was conducted in three steps. The first step includes addition of 1 ml from NaOH, SDS, and NaHCO_3_ at concentration of 5X (+1 or −1) to 1 ml water and 1 ml of the fungal suspension (0.5 gm of the fungal mat/ml) to get a final concentration of 1X of each used chemical compound. Then, mixture was incubated for 30 min at room temperature. The second step includes H_2_O_2_ addition at concentration of 2X (+1 or −1) to 1 ml of the treated fungal suspension from step 1 to reach final concentration of 1X of H_2_O_2_. The mixture was then incubated for 1 min at room temperature.

After each abovementioned treatment step, the supernatant was collected by centrifuging the treated fungal suspension at 6000 rpm. Subsequently, the fungal pellet was washed using 1X phosphate buffered saline (PBS) (alternatively common saline solution can be used). In the third step, the cell pellets were washed using 60% ethanol and left at room temperature for 20 min. After each abovementioned washing step and centrifugation, the supernatant was preserved to determine the amount of the released protein and DNA.

### 2.4. Fungal Ghost Cells Evaluation Using a Light Microscope

The fungal ghost cells sample from each fungus was examined by using a light microscope. The quality of the prepared ghost cells has been determined based on the cellular structure as being either intact or deformed, and then, the overall fungal ghost quality is given as %.

### 2.5. Determination of DNA Concentration

The concentration of DNA in the supernatant after each ghost cells preparation step for each fungus was determined by measuring the absorbance at 260 nm. A quartz cuvette was used. An extinction 260 = 1 corresponds to 50 *μ*g dsDNA ml^−1^ [27].

### 2.6. Determination of Protein Concentration

Concentration of released protein from each ghost cells preparation step for each fungus (the different supernatants) was determined by spectrophotometry at 280 nm. A quartz cuvette was used. The protein concentration was derived from the bovine serum albumin (BSA) standard curve [14].

### 2.7. Detection of Fungal Viability

Fungal ghost preparations of both *A. flavus* and *A. niger* were investigated for the possibility of the presence of any viable cells by subjecting them to growth on Sabouraud's dextrose agar plates at 28°C for 7 days.

### 2.8. Transmission Electron Microscopy for Examination of *A. flavus* and *A. niger* Ghost Cells

A transmission electron microscope (JEOL TEM 100 CX) was used for the examination of *A. flavus* and *A. niger* ghost cells.

### 2.9. In Vitro Tissue Culture of Jojoba (*Simmondsia chinensis* (Link) Schn.)

*In vitro* tissue culture of jojoba was prepared as previously described by Mohasseb et al. [28]. The plant cultures were cultivated in jars and maintained in diffuse light (1000–2000 lx), 16 h photoperiod at 25 ± 2°C, and 50–60% relative humidity in a Tissue Culture Unit (Pharmaceutical Bio-Products Research Department, Genetic Engineering and Biotechnology Research Institute, City of Scientific Research and Technological Applications). Jojoba culture jars used in this study were incubated after infection or infection followed by treatment separately from our usual culture establishment for the *in vitro* plant tissue culture to avoid spread of fungal contamination throughout the system.

### 2.10. Infection of Jojoba Tissue Culture with *A. flavus* or *A. niger* and Development of a Fungal Control Trial

Tissue culture of jojoba was infected by spraying with *A. flavus* or *A. niger* (10^3^ CFU) representing the phytopathogenic fungi. After 24 hr, the plant tissue culture was sprayed with different FGCCC of SDS, NaOH, and NaHCO_3_ for each of *A. niger* and *A. flavus* for 20 min followed by treatment with (b) H_2_O_2_ FGCCC solution for 20 min. Then, the treated plants were sprayed with sterile distilled water. Infected jojoba tissue culture without treatment was used as negative control experiment. The fungal growth was observed after 5 and 10 days of infection or infection followed by treatment.

## 3. Results and Discussion

It is obvious that phytopathogenic fungi result in several and diverse problems associated with a potential vast impact on plant production. Phytopathogenic fungi, thus, have a huge negative part in providing food efforts for the ever-growing world population. The diseases they cause in plants, as well as the mycotoxins they produce, severely threaten agricultural production. Much effort has been made to minimize damage caused by phytopathogenic fungi in agricultural setups including various management/control approaches and innovations. Fungal plant pathogens have been controlled by several approaches such as inducing plant natural resistance (using plant defence molecules in agricultural production) against invading fungi [29], fungicides [30], biological pesticides [31], and using plant extracts [8].

The sponge-like protocol was primarily designed to use cheap and safe chemical compounds (NaOH, SDS, NaHCO_3_, and H_2_O_2_) for microbial ghost cells preparation [14]. Ghosts from various microbes were produced by this gentle chemical protocol that was applied to induce evacuation-pores at the microbial cell wall [14–17, 19–26]. The main feature that distinguishes this protocol from other chemical evacuation methods was the combined use of MIC and MGC of the used chemicals responsible for killing microbes according to the optimal experimental design to prepare microbial ghosts mapped by full or reduced Plackett–Burman experimental design. MIC of a used chemical will kill microbial cell while MGC allows cells to escape and live in the presence of a chemical compound, but still it has an effect on the cell wall. The MIC of NaOH gently induces the pore (s) in the microbial cell wall, while SDS is essential to wash the “sponge-like” cells content, as well as to perturb and destabilize the cell wall/plasma membrane of yeasts and fungi [32], and NaHCO_3_ increases the cell permeability. Finally, H_2_O_2_ is applied to hydrolyse the remaining nucleic acids in the ghosts, and ethanol is used to inactivate the remaining nonlysed cells [14].

Our scientific group is working extensively to establish the sponge-like protocol and expanding its applications in humans and economically important animal and plant healthcare.

Microbial ghosts prepared by this protocol can be applied in immunization [17, 18], as a drug delivery system [19], and even DNA and protein isolation [25, 26]. In this study, the protocol was applied to protect *in vitro* tissue cultures of plants against fungal pathogens. As a model, tissue culture of jojoba (*Simmondsia chinensis* (Link) Schn.) was infected with *A. flavus* and *A. niger* representing the phytopathogenic fungi. The success of the protocol to prevent plant fungal infection can be developed to be introduced in field applications or research purposes (via spraying or immersing the plants in field or tissue cultures with chemical compounds involved in the study at concentrations achieving the best conditions for fungal cells killing and ghosts production).

The best conditions for *A. flavus* and *A. niger* ghosts production previously mapped [25, 26] by randomization experimental design (reduced Plackett–Burman experimental design) were used to prepare fungal ghosts. SDS, NaOH, NaHCO_3_, and H_2_O_2_ were used in their MIC (+1 level) or MGC (−1 level) according to this determined optimal experimental design for each fungus. After each step of fungal ghost cells preparation, the release of both of DNA and protein from the fungal cells was evaluated spectrophotometrically at 260_nm_ and 280_nm_, respectively, as presented in Table 2.

The quality of the prepared ghost cells has been determined using a light microscope based on the cellular structure as being either intact or deformed, and then, the overall fungal ghost quality was given as % (Table 2).

**Figure 1:**
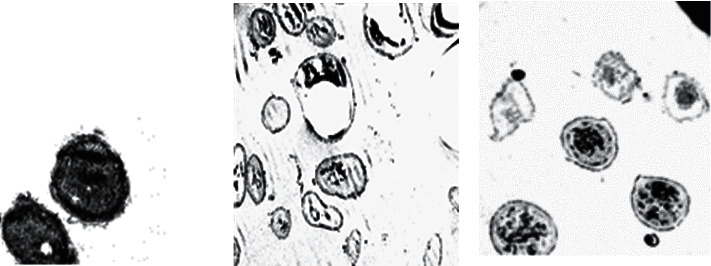
Transmission electron microscope images indicating evacuation and morphological changes in appearance of formed *A. flavus* and *A. niger* ghost cells (magnification at 2000X). (a) *A. flavus* control cells; (b) *A. flavus* ghost and dead cells; and (c) *A. niger* ghost and dead cells.

No growth was obtained on Sabouraud's dextrose agar plates after ghost cells cultivation from either *A. flavus* or *A. niger*, which proves the loss of the fungal cytoplasmic content during the process and the death of these fungi. Fungal cells were killed and well evacuated as proved by the transmission electron microscopy (Figure 1).

After confirmation of preparing high-quality ghost cells, the same conditions were mimicked to control plant fungal infection.

**Figure 2:**
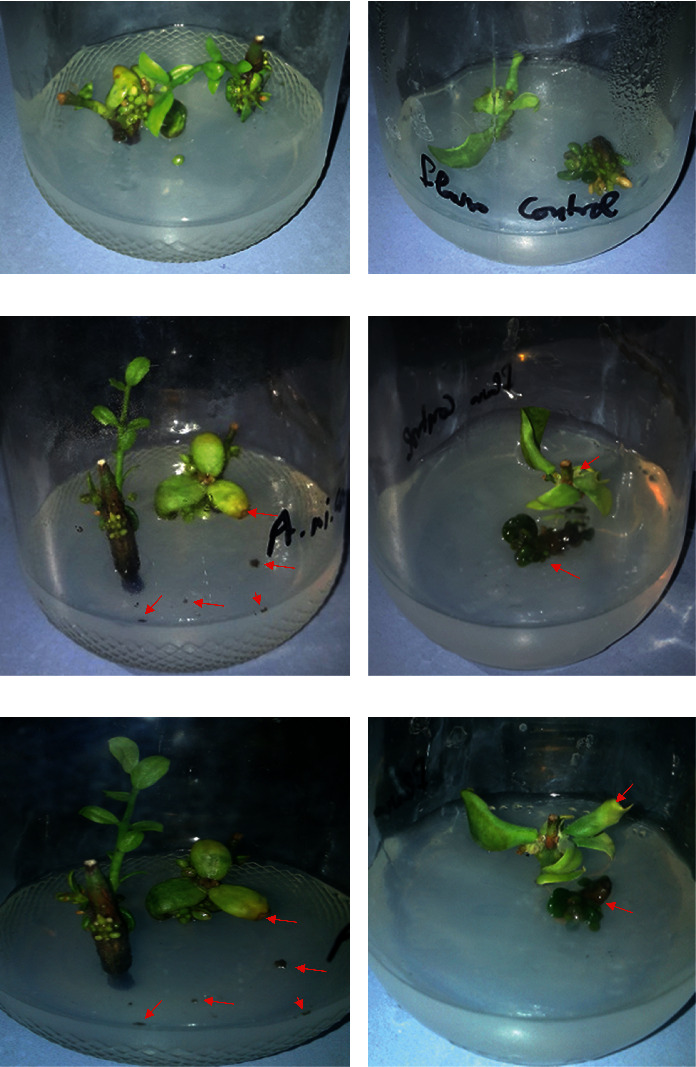
Jojoba grown in tissue culture and infected with either *A. niger* or *A. flavus* cells without any further treatments. (a), (c), and (e) Plant at days 0, 5, and 10, respectively, after spraying with *A. niger* cells. (b), (d), and (f) Plant at days 0, 5, and 10, respectively, after spraying with *A. flavus* cells. Red arrows refer to fungal growth. In case of *A. flavus*, plant nearly stopped growth.

**Figure 3:**
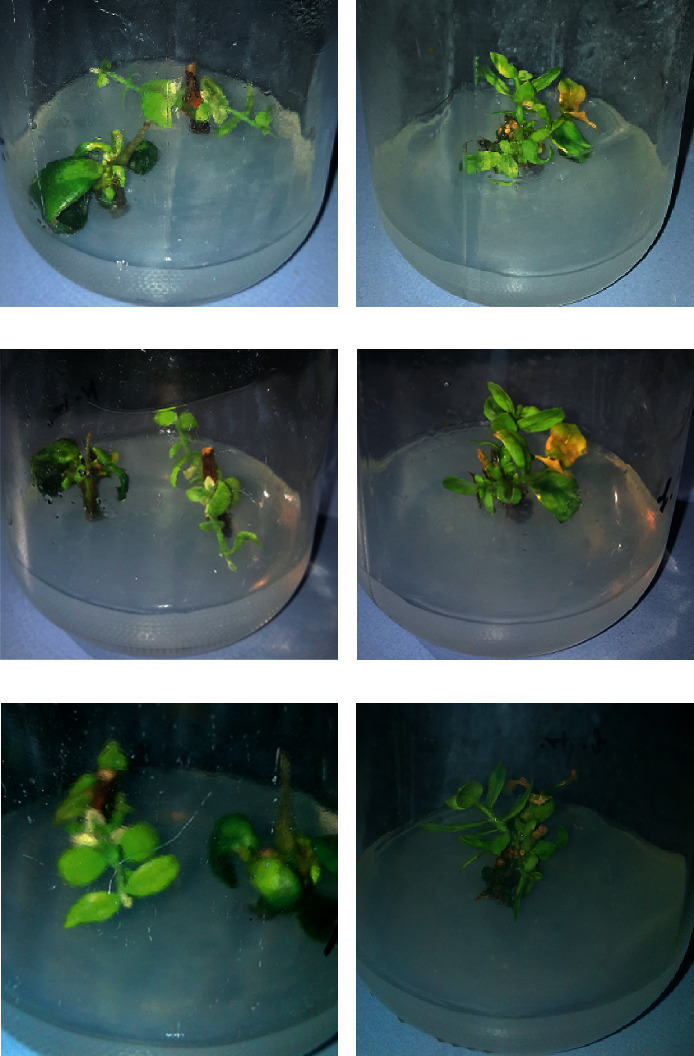
Jojoba grown in tissue culture, infected with either *A. niger* or *A. flavus* cells, and sprayed afterwards with FGCCC.(a), (c), and (e) Plant at days 0, 5, and 10, respectively, after treatment with *A. niger* cells then sprayed with FGCCC. (b), (d), and (f) Plant at days 0, 5, and 10, respectively, after treatment with *A. flavus* cells and then sprayed with FGCCC. Plant continues to grow without any sign of fungal growth on the tissue culture medium or plant parts.

Jojoba grown in tissue culture and sprayed with fungal cells with or without treatment were examined on day 0 (plant grown before any infection or infection followed by treatment), day 5 (plant at day 5 after infection or infection followed by treatment), and day 10 (plant at day 10 after infection or infection followed by treatment). We observed the presence of fungal growth in case of control experiments at days 5 and 10 on the tissue culture medium, as well as plant parts (Figure 2), and the absence of any fungal growth in case of plant infected with fungal cells and treated with FGCCC even at day 10 (Figure 3).

This study succeeded in preventing plant fungal infection via fungal cell evacuation. The used FGCCC inhibits any form of fungal growth. The spraying of fungal cells during infection of plant tissue culture may cause some spores to contaminate the culture medium as seen in negative control experiments, while no sign of fungal growth was observed on the culture medium of treated plants even with multiple spraying steps with fungal cells and FGCCC solutions. These results can be applied through spraying the plants in field or tissue cultures with chemical compounds involved in the study at concentrations achieving the best conditions for fungal cells killing and ghosts production. However, further studies are recommended to screen the optimal incubation time for applying these chemicals to the plant to prevent any negative effect they may cause on plant growth and quality.

## 4. Conclusions

This preliminary study aimed to use sponge-like protocol for preparing FGCCC spray able to protect plants against fungal pathogens. *A. flavus* and *A. niger* FGCCC were calculated for each, and ghost cells were confirmed by measuring the release of each of protein and DNA, as well as by the use of the light and electron microscope. The FGCCCs were examined for their ability to suppress the fungal infection on jojoba tissue culture. No fungal growth was observed in the treated experiments compared with the control. Apparently, the used chemical compounds at the calculated concentrations did not affect the plant growth. This study might be a step towards a new approach to control phytopathogenic fungi. Further studies are in need to validate and to optimize our results.

## Figures and Tables

**Table 1:** The optimal experimental design for ghost cells preparation from each fungus including four chemicals variables in +1 or −1 level.

Fungal strain	NaOH	SDS	NaHCO_3_	H_2_O_2_
*A. flavus*	−1 (0.001 g/ml)	+1 (0.0001 g/ml)	−1 (0.01 g/ml)	+1 (0.15 ml/ml)
*A. niger*	−1 (0.001 g/ml)	+1 (0.0001 g/ml)	+1 (0.3 g/ml)	−1 (0.001 ml/ml)

**Table 2:** Spectrophotometry evaluation of released DNA and protein from ghost cells and percentage of ghost cell quality.

Fungal strain	NaOH/SDS/NaHCO_3_ step		H_2_O_2_ step		Ethanol step		Cell quality (%)
	DNA (*μ*g/ml)	Protein (mg/ml)	DNA (*μ*g/ml)	Protein (mg/ml)	DNA (*μ*g/ml)	Protein (mg/ml)	
*A. flavus*	31.4	0.542	15.65	0.161	1	0.016	80%
*A. niger*	29.45	0.664	0.15	0.001	5.53	0.007	80%

## Data Availability

All the data are given in the original file.
